# New record of *Cyrtonotula* Uvarov, 1939 (Blaberidae, Epilamprinae) from China, with three new species based on morphological and COI data

**DOI:** 10.3897/zookeys.1021.59526

**Published:** 2021-03-02

**Authors:** Yi-Shu Wang, Rong Chen, Du-Ting Jin, Yan-Li Che, Zong-Qing Wang

**Affiliations:** 1 College of Plant Protection, Southwest University, Beibei, Chongqing 400715, China Southwest University Chongqing China

**Keywords:** ABGD, *
Cyrtonota
*, DNA barcodes, habitat, neighbor joining, species identification

## Abstract

The genus *Cyrtonotula* Uvarov, 1939 (Blaberidae, Epilamprinae) is recorded for the first time from Hainan Island, China. Three new species, *Cyrtonotula epunctata* Wang & Wang, **sp. nov.**, *C. maculosa* Wang & Wang, **sp. nov.**, and *C. longialata* Wang & Wang, **sp. nov.**, are described based on morphological data and a molecular analysis using Automatic Barcode Gap Discovery (ABGD). Additional barcode data of blaberid species, including these three new species, are provided to facilitate future species identification. Morphological photographs and habitat photos of these new species, as well as a key to the known species, are provided.

## Introduction

The Epilamprinae genus *Cyrtonota* was proposed by Hanitsch (1929), with *C. lata* as type species, on the basis of one single female specimen from Sumatra. It was characterized by its comparatively large pronotum, hind metatarsus length barely equal to the succeeding joints combined, and the reduced tergum with nearly truncate apex. Owing that the name is preoccupied by a genus of spider (Simon 1864), a replacement name, *Cyrtonotula*, was proposed by Uvarov (1939).

Since then, no more species have been reported from this genus. Roth (2003) removed it from Epilamprinae and treated it as Blaberidae*incerate sedis*. Recently, Mavropulo et al. (2015) returned it back to Epilamprinae as a result of a phylogenetic analysis based on 28S and the male genital characters; this was verified by Djernæs et al. (2020) using four mitochondrial and three nuclear genes. Mavropulo et al. (2015) described two species from Indonesia and provided a description on the male genitalia of this genus for the first time. Later, a fourth species was described from the Philippines (Lucañas 2017). To date, there are four known *Cyrtonotula* species worldwide and none from China.

Species of *Cyrtonotula* are currently identified primarily on the basis of morphological characters, mainly the shortened tegmina and wings, the shape of the pronotum, and male genitalia. DNA barcoding had not been employed to explore the diversity of *Cyrtonotula*.

DNA barcodes (the standard COI sequence) have been proven to be a useful supplementary method in identifying cockroach species and have been effective in resolving problems, such as sexual dimorphism and the identification of nymphs (Che et al. 2017; Bai et al. 2018; Wang et al. 2018). In DNA barcoding studies of cockroaches, the Automatic Barcode Gap Discovery (ABGD) method of species delineation (Puillandre et al. 2012) is widely used and has proven effective in discerning cockroach species (Bai et al. 2018; Wang et al. 2019; Li et al. 2020). Here, *Cyrtonotula* is reported from China, and three new species are described, with the aid of DNA barcoding.

## Material and methods

### Morphological study

Type specimens are deposited in the Institute of Entomology, College of Plant Protection, Southwest University, Chongqing, China (**SWU**). Male genital segments were processed with 10% NaOH for maceration of the soft tissues, observed in glycerol with a Motic K400 stereomicroscope or a Leica M205A stereomicroscope, and preserved with the remainder of the specimen in ethyl alcohol. Photographs were taken with a Leica DFC digital microscope camera attached to a Leica M205A stereomicroscope. All photos and images were processed with Adobe Photoshop CS6. Species descriptions are based on the holotype male. Measurements are given according to the whole sample studied for the description. Sclerites in male genitalia are named according to Klass (1997). The terminology of venation follows Li et al. (2018). Vein abbreviations in this article are as follows:

**ScP** subcosta posterior;

**R** radius;

**RA** radius anterior;

**RP** radius posterior;

**Pcu** postcubitus;

**M** media;

**CuA** cubitus anterior;

**CuP** cubitus posterior;

**V** vannal.

### DNA extraction, amplification, and sequencing

We used standard methods to sample cytochrome c oxidase subunit I (COI) of four species (Table 1) as follows. Total DNA was extracted using Hipure Tissue DNA Mini Kit from the hind legs of alcohol-preserved specimens according to the standard DNA barcoding methods for the cockroach. The mitochondrial COI gene was amplified by PCR using primer sets of COI-F3 (5'-CAACYAATCATAAAGANATTGGAAC-3') and COI-R3 (5'-TAAACTTCTGGRTGACCAAARAATCA-3') resulting in a fragment length of 658 bp for genetic analysis after trimming the primers from the amplified product. The amplification reaction was performed in a total volume of 25 µL, including 23 µL T3 DNA polymerase, 1 µL of each primer and 1 µL DNA template. The thermal cycling conditions were as follows: initial denaturation of 2 min at 98 °C followed by 35 cycles of denaturation at 98 °C for 10 s, annealing at 53 °C for 10 s, extension at 72 °C for 10 s, and a final extension at 72 °C for 5 min; the samples were then held at 8 °C. The amplified samples were evaluated in 1% agarose gels. Sequencing in both directions was performed by BGI Technology Solutions Co. Ltd (BGI-Tech) (Beijing, China).

**Table 1.** Samples of COI genes used in this study.

Genus	Species	Voucher number	Sequence ID	Locality (China)	Accession number
* Opisthoplatia *	*O. orientalis*	C01.1M	OpisOrie03	Guangzhou, Guangdong	MW649981
		C01.3M	OpisOrie05	Wuzhishan, Hainan	MW649982
* Cyrtonotula *	*C. epunctata* sp. nov.	M01.1M	CyrtTest01	Diaoluoshan, Hainan	MW649978
		M01.2F	CyrtTest02	Diaoluoshan, Hainan	MW649979
		M01.3F	CyrtTest03	Wuzhishan, Hainan	MW649980
	*C. maculosa* sp. nov.	K01.1M	QuadBrac01	Yinggeling, Hainan	MW649972
		K01.2M	QuadBrac02	Yinggeling, Hainan	MW649973
		K01.3F	QuadBrac03	Yinggeling, Hainan	MW649974
	*C. longialata* sp. nov.	K02.1F	QuadMacr01	Baoting, Hainan	MW649975
		K02.2M	QuadMacr02	Dalimuling, Hainan	MW649976
		K02.3M	QuadMacr03	Bawangling, Hainan	MW649977
	cf. *Cyrtonotula* sp. MNHN BL13				KY497672
* Pseudophoraspis *	*P. kabakovi*				MH755938
					MH755939
* Rhabdoblatta *	*R. densimaculata*				MK547402
					MK547405
					MK547406
	*R. mascifera*				MK547407
					MK547408
Outgroup	*Mantis religiosa*				KR148854

### Sequence processing and phylogenetic analyses

A total of 11 mitochondrial COI sequences were obtained from four Epilamprinae species, plus one *Cyrtonotula* sequence, another seven Epilamprinae sequences, and one sequence representing the mantis outgroup were downloaded from NCBI for phylogenetic analyses (Table 1). Sequences were aligned in online MAFFT 7 (https://mafft.cbrc.jp/alignment/server/) using the Q-INS-i algorithm. The alignment was then manually corrected in MEGA 7 (Kumar et al. 2007). Intraspecific and interspecific genetic distances are quantified based on the Kimura 2-parameter (K2P) distance model (Kimura 1980) in MEGA 7. The neighbor joining (NJ) tree was constructed in MEGA 7 under the Kimura 2 parameter model (K2P). Statistical support was estimated with 1000 bootstrap replicates. To determine putative species in our study, we used the species delimitation approach, Automatic Barcoding Gap Discovery (ABGD), which was performed using the online webserver (http://wwwabi.snv.jussieu.fr/public/abgd/). The settings were as follows: Pmin = 0.001, Pmax = 0.1, Steps = 10, X (relative gap width) = 1.0, Nb bins = 20, and using Jukes Cantor (JC69) distance.

## Results

### Species delimitation based on COI and morphological data

**Figure 1.**
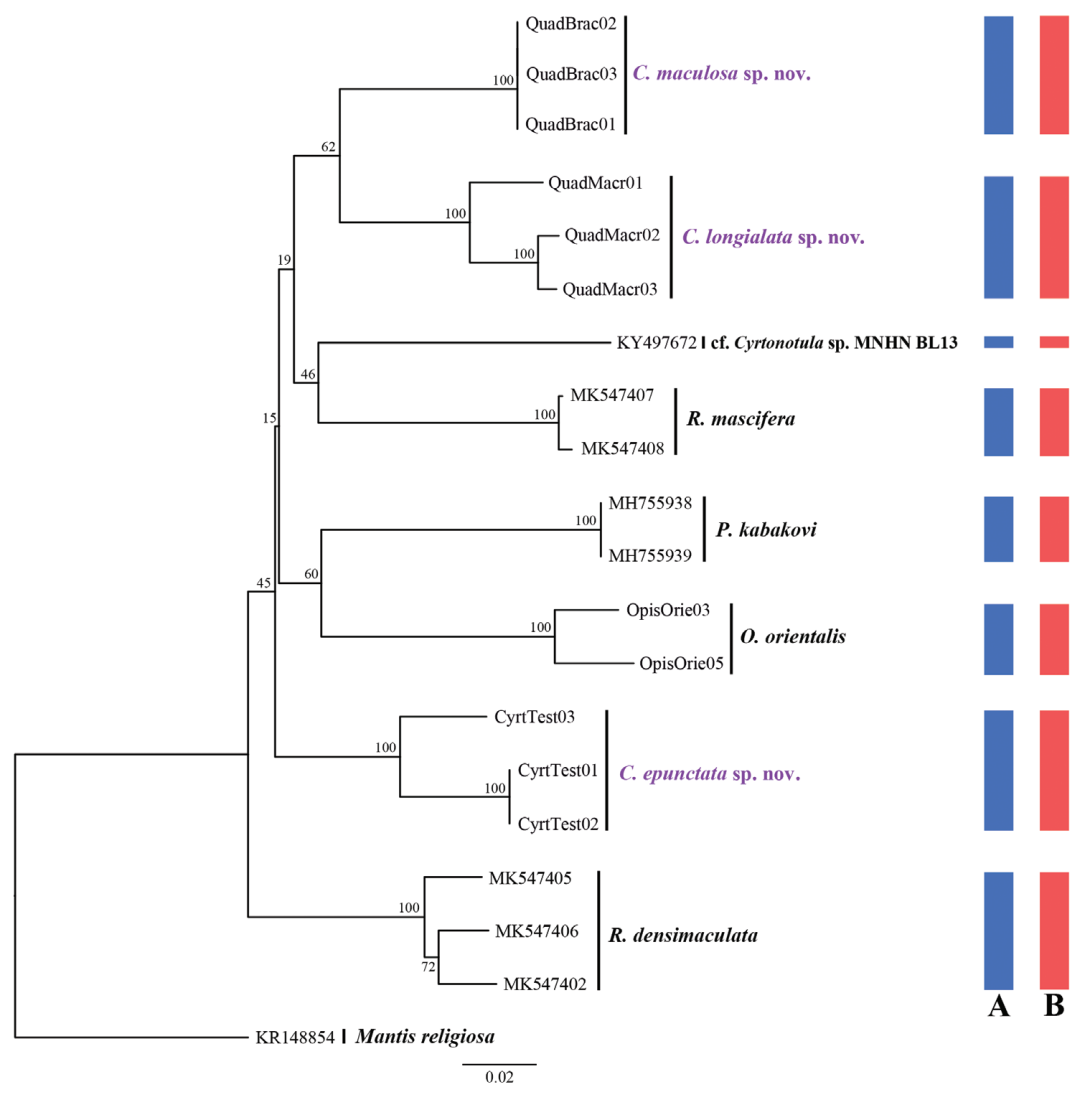
Neighbor joining (NJ) tree derived from COI sequences based on the K2P model. Morphospecies blue; MOTUs in ABGD red.

In this study, we acquired 11 COI sequences representing three *Cyrtonotula* and one *Opisthoplatia* species. All new sequences were deposited in GenBank (accession numbers MW649972 to MW649982 in Table 1). An NJ analysis revealed that clades from the same species, including male and female samples, constituted monophyletic groups with high support values (Fig. 1). We observed the lowest and highest K2P interspecies genetic distance among these species, 0.1056 for *C. maculosa* sp. nov. and *C. longialata* sp. nov., and 0.1367 for *C. epunctata* sp. nov. and *C. maculosa* sp. nov. We used the ABGD method to delimit *Cyrtonotula* species. Three MOTUs were detected in the ABGD analysis, which are completely consistent with the results based on morphological characters (Fig. 1) for *C. epunctata* sp. nov., *C. maculosa* sp. nov., and *C. longialata* sp. nov.

## Systematics

### Cyrtonotula

Taxon classificationAnimaliaBlattodeaBlaberidae

Uvarov, 1939

3327F5BE-0571-5F7F-9C55-A792D4F90281

Cyrtonota Hanitsch 1929: 281. Type species: Cyrtonota lata Hanitsch, 1929.
Cyrtonotula Uravov 1939: 459, replacement name for Cyrtonota Hanitsch, 1929; Princis 1967: 662; Mavropulo et al. 2015: 18; Lucañas 2017: 132. New record from China.

#### Diagnosis.

Medium-sized cockroaches. Both sexes similar. Ocular distance slightly narrower than the distance between antennal sockets, greater than ocellar distance. Pronotum broad, anterior margin curved and posterior margin obtusely produced. Tegmina and wings usually brachypterous, not reaching the abdominal apex (except for macropterous *C. longialata* sp. nov.), their apices somewhat rounded or approximately truncated. Anteroventral margin of front femur Type B; tarsi moderately long; hind metatarsus slender, distinctly longer or nearly equal to the remaining segments combined, armed with two or less equal rows of spines and large apical pulvilli; succeeding tarsomeres armed only with spines surrounding the large pulvilli; the pretarsus with arolium, claws symmetrical and unspecialized. Supra-anal plate entire, with a median incision. Cerci elongate. Subgenital plate large, nearly symmetrical or somewhat asymmetrical. Styli cylindrical.

Male genitalia. Right phallomere Morphnini-type (Anisyutkin and Yushkova 2017): consisting of sclerites R1T, R2, R3, R4, and R5; R4 irregular plate-like, separated; R3 connected to R5. The shape of apical sclerite of L2D irregular and variable. Sclerite L3 hook apically blunt; the folded structure distinct with bristles (visible at high magnification), sclerite L4U present.

#### Remarks.

Based on the closely similar structure of right phallomere in the epilamprines, four genera have been recorded from China: *Morphna* Shelford, *Pseudophoraspis* Kirby, *Rhabdoblatta* Kirby, and *Stictolampra* Hanitsch (Beccaloni 2014; Anisyutkin and Yushkova 2017).

The genus *Cyrtonotula* differs from *Rhabdoblatta*, *Pseudophoraspis*, and *Stictolampra* principally by its reduction of the tegmina and wings. Additionally, *C. longialata* sp. nov. is morphologically somewhat similar to some *Rhabdoblatta* and *Stictolampra* species but can be distinguished by the presence of glandular specialization on the abdominal tergites, basal portion of sclerite L2D, and the non-punctate pronotum.

The genus *Cyrtonotula* can be distinguished from *Morphna* by the structure of hind tarsi: metatarsus distinctly longer or about as long as other segments combined, with relatively numerous tarsal spines (metatarsus slightly shorter or nearly equal to remaining segments combined with larger pulvilli, tarsal spines few or absent).

### Key to species of *Cyrtonotula* worldwide

**Table:** 

1	Tegmina and wings fully developed, both extending beyond the abdominal apex	***C. longialata* sp. nov.**
–	Tegmina and wings reduced, not reaching the abdominal apex	**2**
2	Pronotum testaceous without maculae	***C. epunctata* sp. nov.**
–	Pronotum with scattered maculae	**3**
3	Vertex with 3 longitudinal dark lines	***C. lata* Hanitsch**
–	Vertex with longitudinal lines fewer than 3	**4**
4	Front femur Type B_1_	***C. maquilingensis* Lucañas**
–	Front femur Type B_2_	**5**
5	First abdominal tergum specialized, sclerite L3 comparatively truncated at apex	***C. maculosa* sp. nov.**
–	Abdominal tergites unspecialized, sclerite L3 with apex rounded to truncated	**6**
6	Vertex with yellowish striation, tegmina and wings strongly reduced	***C. tertia* Mavropulo, Anisyutkin, Zagoskin, Zagoskina, Lukyantsev & Mukha**
–	Vertex speckled with black, tegmina and wings weakly reduced	***C. secunda* Mavropulo, Anisyutkin, Zagoskin, Zagoskina, Lukyantsev & Mukha**

### Cyrtonotula epunctata

Taxon classificationAnimaliaBlattodeaBlaberidae

Wang & Wang sp. nov.

4821DB9B-9A01-5B30-8CD4-CE9023250C2A

http://zoobank.org/1B3A01E3-2D33-4263-A61F-656D79F82863

Figs 2A–L , 5A

#### Type material.

***Holotype.*** China • male; Hainan Prov., Lingshui County, Diaoluoshan Mountain; 916 m; 16 Apr. 2015; Lu Qiu & Qi-Kun Bai leg.; SWU-B-BB100101.

***Paratypes*.** China • 1 male & 2 females; same collection data as holotype; 18 Apr. 2015; SWU-B-BB100102 to 100104 • 1 female; Hainan Prov., Wuzhishan Nature Reserve; 795 m; 18 May 2014; Xin-Ran Li, Shun-Hua Gui & Jian-Yue Qiu leg.; SWU-B-BB100105 • 1 female; Hainan Prov., Diaoluoshan Mountain; 275 m; 25 May 2014; Xin-Ran Li, Shun-Hua Gui & Jian-Yue Qiu leg.; SWU-B-BB100106.

#### Differential diagnosis.

The new species readily differs from all its congeners in the spination of hind tarsi. *Cyrtonotula epunctata* sp. nov. resembles *C. lata* Hanitsch, 1929 in testaceous body color and the length of hind metatarsus, but the new species can be distinguished from *C. lata* by the following characters: the coloration of facial part black, with clypeo-labral area yellowish brown, and vertex without visible lines (vs deep testaceous and vertex with three longitudinal dark lines in *C. lata*), and tegmina only reaching to the posterior margin of the third abdominal segment (vs reaching over the sixth abdominal tergite in *C. lata*).

#### Description.

***Measurements* (mm).** Overall length: male 20.7–21.0, female 28.9–39.5; pronotum length × width: male 6.3–6.5 × 9.3–9.5, female 8.5 × 11.3; tegmen length: male 9.3–9.6 × 5.6–5.9, female 13.0–18.6 × 8.0–11.9.

**Figure 2.**
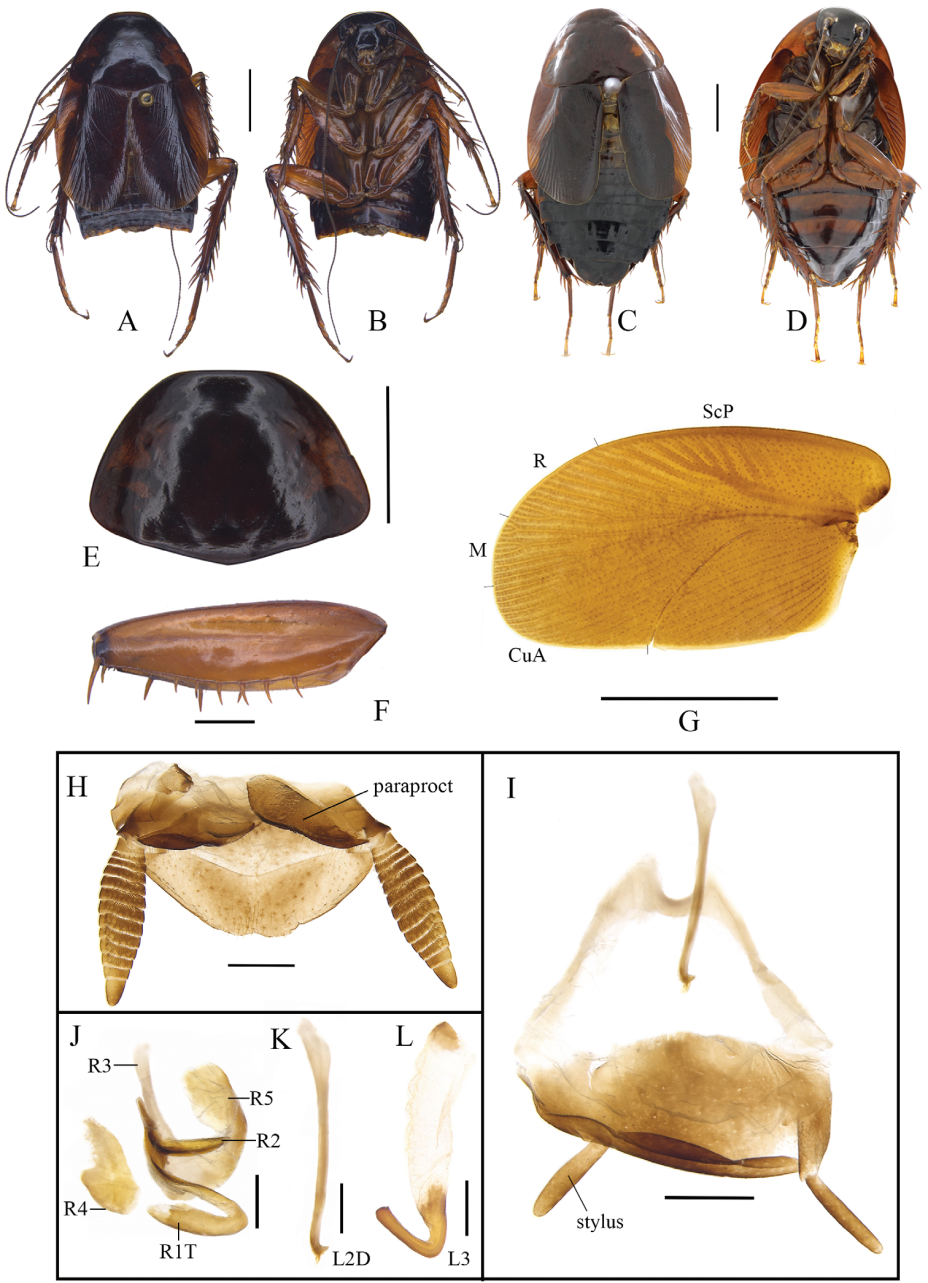
*Cyrtonotula epunctata* Wang & Wang, sp. nov. **A, B, E–L** male **C, D** female **A** paratype, dorsal view **B** paratype, ventral view **C** paratype, dorsal view **D** paratype, ventral view **E** pronotum, dorsal view **F** front femur, ventral view **G** tegmen **H** supra-anal plate, ventral view **I** subgenital plate, dorsal view **J** right phallomere, dorsal view **K** median phallomere (sclerite L2D), dorsal view **L** left phallomere (sclerite L3), dorsal view. Scale bars: 1.0 cm (**C, D**); 5.0 mm (**A, B, E, G**); 1.0 mm (**F, H, I**); 0.5 mm **(J–L)**.

**Male.** Colouration testaceous. Surfaces smooth and glossy (Fig. 2A). Eyes black. Ocellar spots yellow white. Vertex, frons black; clypeus and labrum yellowish brown (Fig. 2B). Pronotum deep testaceous, without spots (Fig. 2E). Tegmina auburn, moderately punctured (Fig. 2G). Legs ferruginous. Abdomen and cerci dark brown (Fig. 2B).

Vertex concealed. Interocular space same as the width between the antennal sockets, slightly greater than ocellar distance. Pronotum nearly semicircular, anterior margin parabolic, posterior margin obtusely angled (Fig. 2E). Tegmina reduced, reaching up the 4^th^ abdominal tergite only; apex rounded; venation distinct, all main veins (*Sc*, *R*, and *CuP*) present, *Sc* thickened (easily visible on ventral side of tegmen) (Fig. 2A, G). Wings vestigial, only reaching to the posterior margin of the 3^rd^ abdominal segment, completely covered by tegmina. Front femur Type B_2_ (Fig. 2F). Hind metatarsus depressed-cylindrical, nearly equal to the succeeding segments combined, with single complete row of spines along ventral margin and several additional spines on inner side; four proximal tarsomeres with pulvilli terminal, the one on the second tarsomere occupying practically the whole length of the segment; claws symmetrical and simple; arolium present (Fig. 5A). Abdominal tergites unspecialized; knobs along the posterior margin indistinct; weak spiracle-bearing outgrowths of tergite VIII with distinct spiracle. Supra-anal plate with the posterior margin widely rounded and a weak mesal incision. Cerci distinctly segmented, densely covered with bristles. Paraprocts of blaberid type, asymmetrical (Fig. 2H). Subgenital plate rounded, slightly asymmetrical; the base of the inner plate bifurcated. Styli cylindrical, apically rounded (Fig. 2I).

**Male genitalia.** Right phallomere with caudal part of sclerite R1T rectangular in shape; cranial part of R1T more or less straight; R2 curved; R3 long; R4 irregular plate-like; R5 large, fused with sclerite R3 in caudal part (Fig. 2J). “chaetae-bearing membranous area” absent. Sclerite L2D not divided into basal and apical parts, slender and rod-like, with basal end tapering and a bifurcated outgrowth born near the basal end (Fig. 2K). Sclerite L3 hooked, apex slightly rounded, with a small tooth on the inner margin less distinct; folded structure present, with bristles. Sclerite L4U distinct (Fig. 2L).

**Female.** Similar to the male but body somewhat larger.

#### Etymology.

Derived from the Latin word *epunctatus*, referring to the lack of visible spots on the body.

### Cyrtonotula maculosa

Taxon classificationAnimaliaBlattodeaBlaberidae

Wang & Wang sp. nov.

18349DCF-071B-5629-810D-1C91AB4A2936

http://zoobank.org/4DC34DE6-DFC6-4F5D-AE5C-B0A35CD8F97A

Figs 3A–L , 5B , 6A

#### Type material.

***Holotype*.** China • male; Hainan Prov., Yinggeling Nature Reserve, Nanfa Conservation Station; 650 m; 21 Apr. 2015; Lu Qiu & Qi-Kun Bai leg.; SWU-B-BB090101.

***Paratypes*.** China • 11 males & 11 females; same collection data as holotype; SWU-B-BB090102 to 090123.

#### Differential diagnosis.

This new species is closely related to *C. tertia* Mavropulo, Anisyutkin, Zagoskin, Zagoskina, Lukyantsev & Mukha, 2015 in the shape of tegmina and body color, but the former can be distinguished from the latter by the specialized abdominal terga (vs unspecialized) and the shape of sclerite L3 of male genitalia, in which L3 hook is comparatively robust and posteriorly truncate distinctly (vs comparatively slender and rounded apically in *C. tertia*).

#### Description.

***Measurements* (mm).** Overall length: male 22.5–27.0, female 31.0; pronotum length × width: male 5.5–6.4 × 7.8–8.2, female 6.3 × 9.1; tegmen length: male 20.6–21.8 × 7.9–8.3, female 25.7 × 9.3.

**Figure 3.**
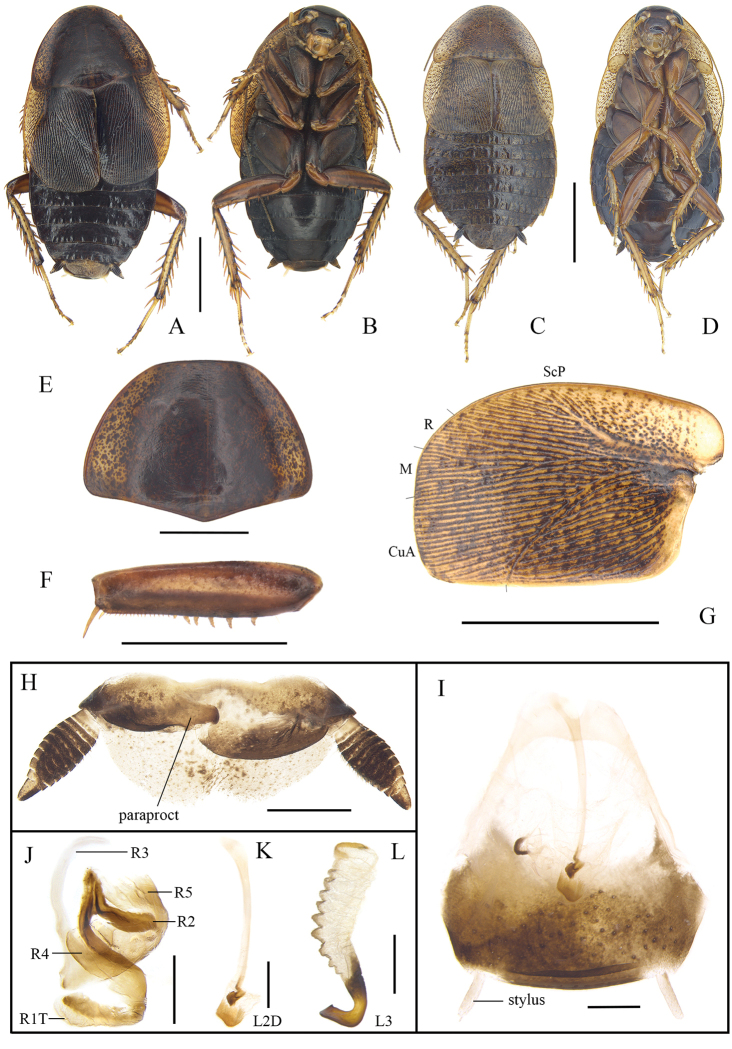
*Cyrtonotula maculosa* Wang & Wang, sp. nov. **A, B, E–L** male **C, D** female **A** holotype, dorsal view **B** holotype, ventral view **C** paratype, dorsal view **D** paratype, ventral view **E** pronotum, dorsal view **F** front femur, ventral view **G** tegmen **H** supra-anal plate, ventral view **I** subgenital plate, dorsal view **J** right phallomere, dorsal view **K** median phallomere (sclerite L2D), dorsal view **L** left phallomere (sclerite L3), dorsal view. Scale bars: 1.0 cm (**C, D**); 5.0 mm (**A, B, E–G**); 1.0 mm (**H–L**).

**Male.** Body yellowish brown (Fig. 3A). Eyes black. Ocellar spots light yellow. Head black except for yellowish brown clypeo-labral area, facial part of head with weak transverse wrinkles (Fig. 3B). Pronotum yellow-brown, with densely scattered irregular brown spots (Fig. 3E). Tegmina dark yellow, punctured, with brown patches spreading along the veins (Fig. 3G). Coxa, trochanter, and femur yellowish brown; tibia and tarsomere light yellow. Abdomen and cerci blackish brown (Fig. 3B).

Vertex slightly exposed, with two longitudinal yellowish-brown bands. Distance between eyes approximately equal to the width between the antennal sockets and smaller than ocellar distance (Fig. 3B). Pronotum campaniform, widely rounded along anterolateral margins, posterior margin obtusely angled (Fig. 3E). Tegmina considerably shortened, reaching the third abdominal tergite, apex subtruncate; venation distinct, all main veins (*Sc*, *R* and *CuP*) present, *Sc* thickened (easily visible on ventral side of tegmen) (Fig. 3A, G). Wings vestigial, only reaching the first abdominal tergite. Anterior margin of fore femur Type B_2_, with 6 or 7 spines (Fig. 3F). Hind metatarsus not quite as long as other segments combined with two rows of spines; well-developed pulvilli on all proximal tarsomeres; claws symmetrical and simple; arolium present (Fig. 5B). Abdominal tergite 1^st^ specialized, lip-like (Fig. 6A); terga 3^rd^–7^th^ with a few knobs (Fig. 3A); spiracles large, located on the posterolateral angles of tergite 8^th^. Supra-anal plate with the posterior margin widely rounded, a weak incision at middle. Cerci fusiform, traces of segmentation distinct. Paraprocts of blaberid type, asymmetrical (Fig. 3H). Subgenital plate entire with hind margin rounded; base of the inner plate bifurcated. Styli cylindrical (Fig. 3I).

**Male genitalia.** Right phallomere with caudal part of sclerite R1T nearly rectangular in shape, cranial part of R1T curved; R2 rounded; R3 elongate apically, curved inward, fused with sclerite R5; R4 plate-like, separated. Sclerite L2D divided into basal and apical parts, basal part rod-like, widened apically, with irregular apical outgrowth; apical part with fine bristles; apical membrane covered with chaetae (Fig. 3K). Sclerite L3 hooked, apically subquadrate; inner margin with a tooth-shaped convexity at apex; folded structure distinct, with bristles; Sclerite L4U present, comparatively narrow (Fig. 3L).

**Female.** Similar to the male. Body color lighter. Tegmina only reaching the second abdominal tergite, with apex distinctly truncated. Abdominal tergites unspecialized.

#### Etymology.

Derived from the Latin word *maculosus*, referring to the scattered with dense spots pronotum and tegmen.

### Cyrtonotula longialata

Taxon classificationAnimaliaBlattodeaBlaberidae

Wang & Wang sp. nov.

58743CE3-1C27-59F2-BC13-3EC14C052D7D

http://zoobank.org/164B8791-1167-4341-BACC-39F40F0B1A5C

Figs 4A–K , 5C , 6B

#### Type material.

***Holotype*.** China • male; Hainan Prov., Limuling Mountain; 18 Apr. 2015; Xin-Ran Li & Zhi-Wei Qiu leg.; SWU-B-BB090201.

***Paratypes*.** China • 3 males; same collection data as holotype; SWU-B-BB090202 to 090204 • 6 males & 2 females; Hainan Prov., Baoting County, Maogan Township; 549–776 m; 11–12 Apr. 2015; Xin-Ran Li, Lu Qiu, Zhi-Wei Qiu & Qi-Kun Bai leg.; SWU-B-BB090205 to 090212 • 1 male; Hainan Prov., Bawangling Mountain; 600–800 m; 29 Apr. 2015; Lu Qiu & Qi-Kun Bai leg.; SWU-B-BB090213 • 2 males; Hainan Prov., Diaoluoshan Mountain; 275m; 24 May 2014; Xin-Ran Li & Shun-Hua Gui leg.; SWU-B-BB090214 and 090215.

#### Differential diagnosis.

The new species principally differs from all its congeners, except for *C. maculosa* sp. nov., in the presence of abdominal tergal glands. From *C. maculosa* sp. nov., *C. longialata* sp. nov. differs in having the completely developed tegmina and wings extending beyond the abdominal apex, the shape of tergal glands (see description below).

#### Description.

***Measurements* (mm).** Overall length: male 27.0–30.0, female 31.0; pronotum length × width: male 6.2–6.4 × 7.8–8.2, female 6.3 × 9.1; tegmen length: male 23.0–25.0 × 8.6–9.2, female 25.7 × 9.3.

**Figure 4.**
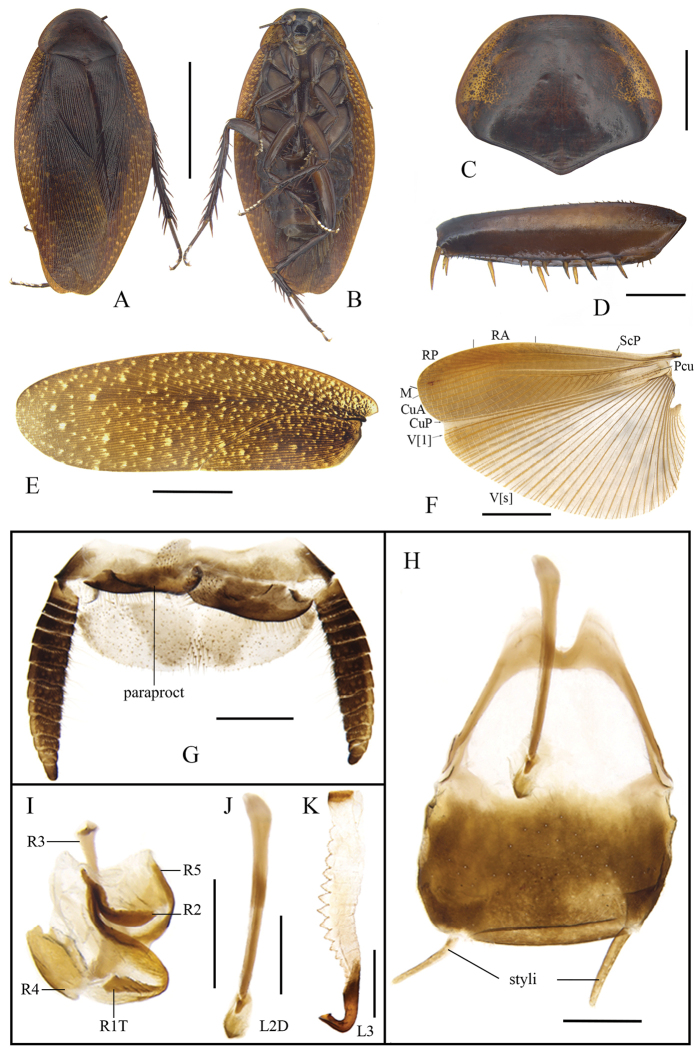
**A–L***Cyrtonotula longialata* Wang & Wang, sp. nov., male **A** holotype, dorsal view **B** holotype, ventral view **C** pronotum, dorsal view **D** front femur, ventral view **E** tegmen **F** wings **G** supra-anal plate, ventral view **H** subgenital plate, dorsal view **I** right phallomere, dorsal view **J** median phallomere (sclerite L2D), dorsal view **K** left phallomere (sclerite L3), dorsal view. Scale bars: 1.0 cm (**A, B**); 5.0 mm (**C, E, F**); 1.0 mm (**D, G–K**).

**Male.** General colour brown (Fig. 4A). Eyes black. Ocellar spots yellow-white. Head black except for brown clypeo-labral area; facial part of head with weak transverse wrinkles and paired impressions under ocelli (Fig. 4B). Pronotum russet, reddish brown at center, speckled with small, brown patches (Fig. 4C). A few yellow spots present in tegmina (Fig. 4E); wings with costal field, radial field, mediocubital field fulvous, and anal field pale brown (Fig. 4F). Legs and abdomen dark yellowish brown. Cerci dark brown (Fig. 4B).

**Figure 5.**
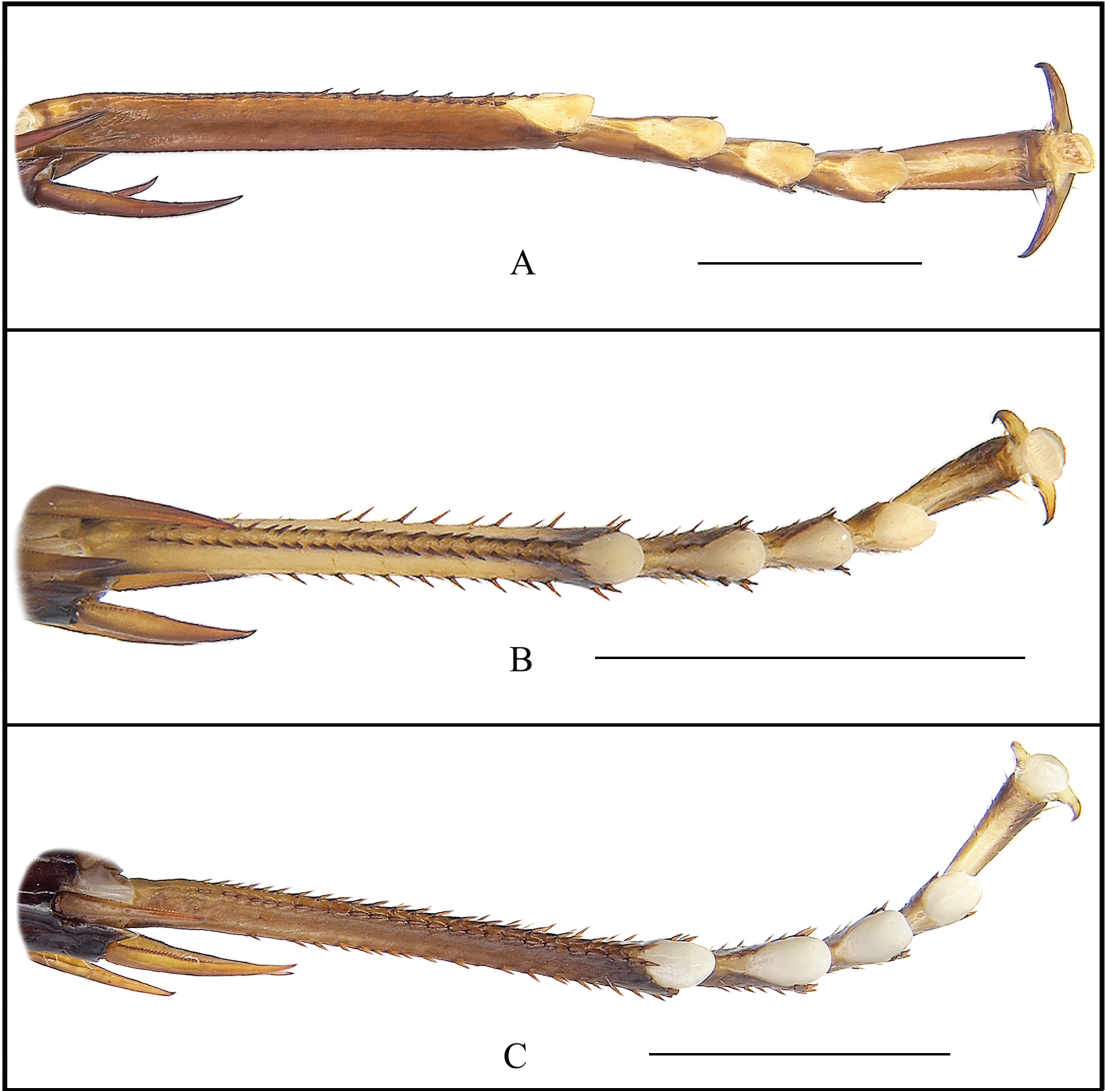
Hind tarsi **A***Cyrtonotula epunctata* Wang & Wang, sp. nov., female, paratype **B***Cyrtonotula maculosa* Wang & Wang, sp. nov., male, holotype **C***Cyrtonotula longialata* Wang & Wang, sp. nov., male, holotype. Scale bars: 2.0 mm.

**Figure 6.**
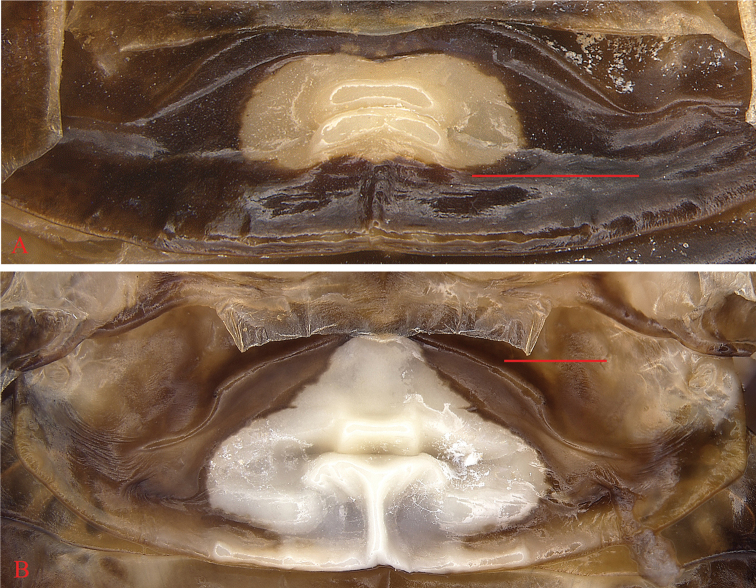
The first abdominal tergal gland **A***Cyrtonotula maculosa* Wang & Wang, sp. nov., male, paratype **B***Cyrtonotula longialata* Wang & Wang, sp. nov., male, holotype. Scale bars: 1.0 mm.

Vertex slightly exposed. Interocular distance as wide as inter-antennal distance, slightly greater than inter-ocellar distance. Pronotum flabellate, widely rounded along anterolateral margins, posterior margin obtusely angled (Fig. 4C). Tegmina and wings completely developed, exceeding abdominal apex; tegmina with rounded apex; venation distinct, all main veins (*Sc*, *R*, and *CuP*) present (Fig. 4E, F). Anterior margin of fore femur B_2_ (Fig. 4D). Hind metatarsus distinctly longer than other segments combined, armed with two rows of spines; pulvilli large on all proximal tarsomeres; claws symmetrical and simple; arolium present (Fig. 5C). The first abdominal tergite specialized, cap-like (Fig. 6B); tergite VIII with posterolateral angles strongly expressed. Supra-anal plate with the caudal margin widely rounded and a weak median incision. Cerci robust, segmented. Paraprocts of blaberid type, asymmetrical (Fig. 4G). Subgenital plate symmetrical, rounded. Base of inner plate bifurcated. Styli long, cylindrical, apically rounded (Fig. 4H).

**Male genitalia.** Right phallomere with caudal part of sclerite R1T nearly rectangular, cranial part of R1T curved; R2 arched; R3 elongate and widened apically, fused with sclerite R5 in caudal part; R4 irregular plate-like, separated (Fig. 4I). Sclerite L2D divided into basal and apical parts, basal part rod-like, widened cranially; apical part trifurcate; apical membrane covered with chaetae (Fig. 4J). Sclerite L3 hooked, apically subtruncate; inner margin with apex pointed; folded structure and bristles present; sclerite L4U distinct (Fig. 4K).

**Female.** Similar to the male. Abdominal tergites unspecialized.

#### Remarks.

Currently, this is the only species of *Cyrtonotula* with fully developed tegmina and wings. This species is placed in *Cyrtonotula* because it closely resembles *C. maculosa* sp. nov. in having sclerite L3 hooked (apex nearly truncate and inner margin with a distinct point) and in the location of tergal gland.

#### Etymology.

The species epithet is derived from the Latin adjective *longialatus*, which refers to the well-developed wings.

**Figure 7.**
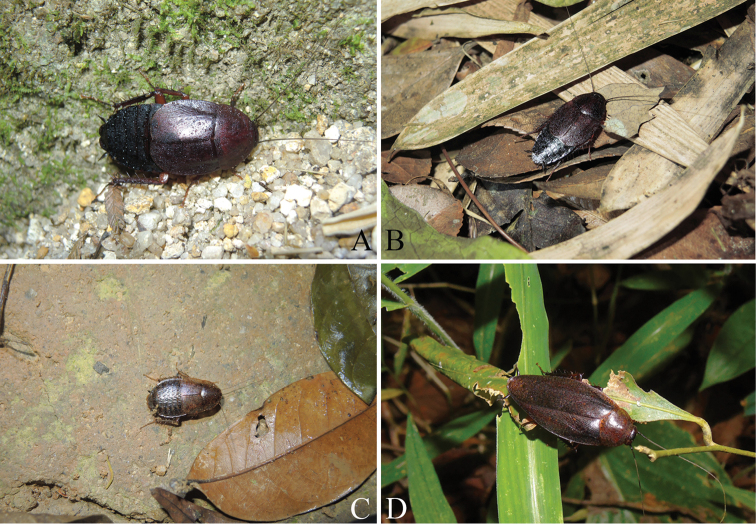
Living *Cytonotula* species from Hainan, China **A** female *C. epunctata* sp. nov. (Diaoluoshan Mountain) **B** male *C. epunctata* sp. nov. (Diaoluoshan Mountain) **C** female *C. maculosa* sp. nov. (Yinggeling Mountain) **D***Cyrtonotula longialata* sp. nov. (Baoting County). Photos: **A–C** by Lu Qiu **D** by Xin-Ran Li.

## Discussion

Flightless cockroaches are usually considered to persist in stable habitats, where food, shelter, and mates are easily accessible (Bell et al. 2007). All of previously known *Cyrtonotula* species are brachypterous, but *C. longialata* sp. nov. is noteworthy for being the first macropterous in this genus. Brachypterous cockroaches, including *C. epunctata* sp. nov. and *C. maculosa* sp. nov., were observed on leaf litter and scree in areas with trickling water (Fig. 7A–C), while *C. longialata* sp. nov., maybe a canopy species, was collected in low vegetation (Fig. 7D). We speculate that habitats of *C. epunctata* sp. nov. and *C. maculosa* sp. nov. may be different from the habitat of *C. longialata* sp. nov., and habitat might be one of the determining factors in variations of the wings. This study is also the first to use COI DNA barcode to evaluate diversity of *Cyrtonotula* species. Our results show that DNA-based species delimitation methods perform well and that individuals were correctly assigned to their corresponding species, although only 10 sequences of *Cyrtonotula* were included here. Therefore, taking into consideration that there are only seven species of the genus found worldwide, we expect more species of *Cyrtonotula*, especially macropterous ones, will be discovered and observed with further sampling, so that the knowledge of this genus *Cyrtonotula* could be comprehensive and explored more deeply.

## Supplementary Material

XML Treatment for Cyrtonotula

XML Treatment for Cyrtonotula epunctata

XML Treatment for Cyrtonotula maculosa

XML Treatment for Cyrtonotula longialata
